# Altered vascular function in chronic kidney disease: evidence from passive leg movement

**DOI:** 10.14814/phy2.14075

**Published:** 2019-04-23

**Authors:** Elissa K. Katulka, Alexandra E. Hirt, Danielle L. Kirkman, David G. Edwards, Melissa A. H. Witman

**Affiliations:** ^1^ Department of Kinesiology and Applied Physiology University of Delaware Newark Delaware; ^2^ Department of Kinesiology and Heath Sciences Virginia Commonwealth University Richmond Virginia

**Keywords:** CKD, endothelial function, flow‐mediated dilation, nitric oxide, PLM

## Abstract

Chronic kidney disease (CKD) is an independent risk factor for the development of cardiovascular disease and is characterized by reduced nitric oxide (NO) bioavailability and vascular dysfunction, typically assessed using brachial artery flow‐mediated dilation (FMD). It has been previously reported that passive leg movement (PLM)‐induced hyperemia, an assessment of lower extremity vascular function, is highly dependent on NO, but has not yet been utilized to assess vascular function in patients with CKD. The purpose of this study was to comprehensively assess vascular function in patients with CKD using PLM, in addition to the traditional FMD technique. Assessment of vascular function via PLM and FMD was performed on 12 patients (CKD, 66 ± 3 years) and 16 age‐matched healthy controls (CON, 60 ± 2 years). Blood velocity and artery diameters during PLM and FMD were measured using duplex ultrasound of the femoral and brachial arteries, respectively. Habitual physical activity, assessed by accelerometry, was performed in a subset of each group. CKD patients had reduced peak leg blood flow (LBF) (384 ± 39 vs. 569 ± 77 mL/min, *P* < 0.05) and change in LBF from baseline to peak (∆peakLBF) (143 ± 22 vs. 249 ± 34 mL/min, *P* < 0.05) during PLM compared to CON. Additionally, PLM responses were significantly associated with kidney function and physical activity levels. As anticipated, FMD was significantly attenuated in CKD patients (5.2 ± 1.1 vs. 8.8 ± 1.2%, *P* < 0.05). In conclusion, both upper and lower extremity measures of vascular function indicate impairment in CKD patients when compared to controls. PLM appears to be a novel and feasible approach to assessing lower extremity vascular function in CKD.

## Introduction

Chronic kidney disease (CKD) is a leading public health concern and is characterized by damage to the kidneys and their inability to effectively filter blood. Reduced kidney function leads to systemic disturbances such as altered blood pressure regulation, acid‐base imbalances, altered fluid and electrolyte regulation, and a buildup of uremic waste products, all of which contribute to organ dysfunction. Moreover, kidney disease is an independent risk factor for the development of cardiovascular disease (CVD) (Sarnak et al. 2003), which is diagnosed in 70% of individuals with CKD over the age of 66 (Saran et al. 2016). Given that individuals with CKD are more likely to die from CVD than progress to end‐stage kidney failure (Saran et al. 2016), it is imperative to better understand the physiological relationship between these two chronic diseases.

Traditional risk factors alone do not sufficiently account for the high prevalence of CVD in CKD (Isbel et al. 2006). Therefore, nontraditional CVD risk factors such as endothelial dysfunction have been identified for their role in the association between the two diseases (Di Lullo et al. 2017). Endothelial dysfunction represents a vascular phenotype which is prone to atherogenesis and serves as a predictive marker of their atherosclerotic disease risk and rate of survival (Bonetti et al. 2003; Deanfield et al. 2007). Endothelial dysfunction is characterized by a reduced bioavailability of nitric oxide (NO), an endogenous signaling molecule produced by endothelial cells, which under homeostatic conditions possesses vasodilatory, antiplatelet, antiproliferative, antiadherent, and anti‐inflammatory properties (Stehouwer 2004; Rajapakse et al. 2015). Impaired formation of NO through the L‐arginine pathway has been established in patients with CKD (Baylis 2008) and is thought to play a role in the pathogenesis of cardiorenal syndrome (Shechter et al. 2009). Reduced NO bioavailability in CKD patients may also occur as a result of increased circulation of uremic toxins such as asymmetric dimethylarginine (ADMA) as well as possible interactions between NO and reactive oxygen species, both of which have been previously identified in this population (DuPont et al. 2011; DuPont et al. 2014). Assessment of the peripheral circulation often provides a feasible approach to characterizing NO‐mediated vascular function and is indicative of vascular risk in the coronary circulation (Anderson and Phillips 2015). Moreover, reduced peripheral endothelial function has been significantly associated with the presence of coronary artery disease in CKD patients (Hirata et al. 2014) and has proven to be highly beneficial to uncovering the mechanisms responsible for the increased incidence of CVD in this population.

It has been previously documented that passive movement of an individual's leg can produce a hyperemic response that is indicative of peripheral vascular function (Wray et al. 2005; Gifford and Richardson 2017). Passive leg movement (PLM) is a newly developed method which directly applies this concept and has been shown to produce a highly NO‐dependent increase in blood flow measurable at the femoral artery (Mortensen et al. 2012; Trinity et al. 2012). In addition, the PLM assessment is relatively simple to conduct, making it an attractive assessment of peripheral vascular function in both healthy and diseased populations (Hayman et al. 2010; McDaniel et al. 2010; Witman et al. 2015; Nelson et al. 2016). Importantly, PLM specifically assesses the lower extremity vasculature, however, to date no previous investigations have evaluated lower limb‐specific vascular function in patients with CKD.

Reduced cardiorespiratory fitness levels are well‐documented in those with CKD (Kirkman et al. 2014). Exercise intolerance is associated with increased rates of disease progression and mortality (Kodama et al. 2009) and may play a role in the elevated risk of CVD identified in this population (Tsai et al. 2017). Use of PLM in CKD is a novel method of assessing endothelial function within the vasculature that is directly responsible for supplying blood and oxygen to the working muscles mainly responsible for locomotion and physical activity. Therefore, PLM may represent localized NO bioavailability and could provide novel mechanistic insight into the association between vascular dysfunction and exercise intolerance in patients with CKD.

Accordingly, the aim of this study was to utilize PLM as a novel tool for the assessment of lower limb‐specific peripheral vascular function in CKD patients and in a group of healthy, aged‐matched controls. In addition, we utilized brachial artery flow‐mediated dilation (FMD), a traditional assessment of vascular function, as a confirmatory indicator of peripheral vascular function. We hypothesized that both PLM and FMD responses would be attenuated in CKD patients when compared to healthy adults, suggestive of reduced endothelial function in both the lower and upper extremities of this population.

## Methods

### Participants

This study was approved by the Institutional Review Board at the University of Delaware. A total of 28 participants (12 CKD patients, 16 healthy age‐matched controls) participated in this study. Patients with CKD and healthy controls were primarily identified through a database of subjects previously enrolled in studies at the University of Delaware and who had provided written consent to being contacted regarding participation in research studies. Additionally, participants were recruited through advertisements located on the University of Delaware campus and in the surrounding Newark, DE region. All participants provided written informed consent prior to participation. Inclusion criteria for patients included stage 3–5 CKD as defined by an estimated glomerular filtration rate (eGFR) of ≤60 mL/min/1.73 m^2^ (calculated using the isotope dilution mass spectrometry‐traceable 4‐variable equation according to the CKD Epidemiology Collaboration [CKD‐EPI]) for a minimum of 3 months (Levey et al. 2009). Patients with CKD were excluded if they required renal replacement therapy, currently used tobacco products, had uncontrolled hypertension, or had any history of autoimmune diseases. In addition, patients were screened for any history of diagnosed CVD, as we sought to identify endothelial dysfunction as it precedes CVD development in those with CKD. Healthy controls were normotensive, nontobacco users, and free of any overt chronic diseases.

### Vascular testing visit

All studies were performed in a temperature‐controlled environment (~23°C). Participants were instructed to report to the laboratory fasted for 12 h, without caffeine, alcohol, or exercise for 24 h prior to the visit, and to avoid taking any medications, vitamins, or supplements on the morning of the visit. Upon arrival to the laboratory, participants underwent intravenous blood sampling, urine sampling, assessment of resting vitals, and body anthropometry. Blood samples were assessed clinically for a complete blood count, complete metabolic panel, and lipid panel. Urine sampling was analyzed clinically for a complete urinalysis. Body composition was assessed using bioelectrical impedance analysis technology (Tanita TBF‐300A, Arlington Heights, IL). Participants then rested supine for a minimum of 20 min prior to vascular function measurements.

### Vascular function protocols

#### Passive leg movement

PLM was performed as previously described (Trinity et al. 2012; Groot et al. 2015; Witman et al. 2015; Rossman et al. 2016) and in accordance with current recommendations (Gifford and Richardson 2017). Briefly, in the upright seated position, the protocol consisted of 60 sec of baseline measurements immediately followed by a 1‐minute bout of passive leg flexion and extension at the knee joint. Duplex ultrasound imaging (Logic e, General Electric Medical Systems, Milwaukee, WI) was achieved using a linear array ultrasound probe (12 Hz) at the common femoral artery, distal to the inguinal crease but above the femoral bifurcation into the superficial and profound femoral branch. Passive movement was achieved by a member of the research team moving the subject's lower leg through a 90° to 180° range of motion at a rate of 1 Hz, while movement cadence was maintained by a metronome. Throughout the duration of the protocol, the unaffected leg remained extended and fully supported. Prior to the start and throughout the duration of the protocol, participants were encouraged not to assist with or resist the leg movement. Additionally, to avoid an anticipatory response, participants were made aware of when the movement phase was within 30 sec of initiation, however, they were not informed of exactly when this movement would begin. Measurement of femoral artery diameter was assessed during baseline, while blood flow velocity was measured throughout the protocol.

#### Brachial artery flow‐mediated dilation

The FMD procedure was performed in accordance with current recommendations (Harris et al. 2010). In short, an inflatable cuff was placed on participants’ upper arm, 2–3 cm proximal to the elbow joint. Brachial artery duplex ultrasound imaging (Logic e, General Electric Medial Systems, Milwaukee, WI) occurred with a linear array ultrasound probe (12 Hz), placed distally to the shoulder joint and proximal to the inflatable cuff. Participants remained supine throughout the duration of the protocol. Following resting baseline measurements, the cuff was rapidly inflated to 250 mmHg for 5 min then rapidly deflated (E20 Rapid Cuff Inflation System, Hokanson, WA). Measurements of brachial artery diameter and blood flow velocity were collected continuously throughout baseline and for 2 min immediately following cuff deflation.

### Habitual physical activity assessment

A subset of CKD (*n* = 8) and healthy control (*n* = 7) participants underwent habitual physical activity assessment through use of wearable accelerometers (ActiGraph L.L.C., Pensicola, FL) for 7 consecutive days. Participants were fitted with accelerometers during the study visit and instructed to wear them for all waking hours over the 7‐day period. Physical activity assessments were completed within 2 weeks of participants’ vascular function assessments. Accelerometer data were analyzed using ActiLife software version 6.11.9. Wear time was validated using the Troiano algorithm (Troiano et al. 2008), and activity variables were calculated using the Freedson Combination 1998 algorithm (Freedson et al. 1998). Activity variables assessed included steps per day, kilocalories of physical activity per day above basal metabolic rate, bouts of sedentary activity per day (“bout” identified as sedentary activity for >10 min), hours spent sedentary per week, and minutes of moderate‐to‐vigorous intensity physical activity per week. Values reported “per day” represent averages of the data collected over the 7‐day period for all subjects.

### Arterial blood flow calculations

Measurements of arterial blood flow velocity and blood vessel diameter were performed in both the PLM and FMD assessments using a Logic e ultrasound system (General Electric Medical Systems, Milwaukee, WI). This system was equipped with a linear array transducer operating at an imaging frequency of 12 MHz. Blood vessel diameters were determined at a perpendicular angle along the central axis of the scanned area. Blood velocities were obtained using the same transducers with a Doppler frequency of 5 MHz. All blood velocity measurements were obtained with the probe appropriately positioned to maintain an insonation angle of 60° or less. The sample volumes were maximized according to vessel size and were centered within the vessel based on real‐time ultrasound visualization. Femoral and brachial arterial diameters were measured and mean velocity (*V*_mean_) values [angle‐corrected and intensity‐weighted area under the curve (AUC)] were then automatically calculated using commercially available software. Using arterial diameter and *V*_mean_, the blood flow in the femoral and brachial arteries were mathematically calculated as follows: blood flow = *V*_mean_ *π* (vessel diameter/2)^2^ × 60, where blood flow is in milliliters per minute.

#### Passive leg movement analysis

Femoral artery blood flow was calculated through offline analysis of anterograde and retrograde blood flow velocities achieved during PLM using continuous ultrasound Doppler imaging. Baseline leg blood flow (LBF) was calculated via 12‐sec averages of anterograde and retrograde blood flow velocities, while second‐by‐second analysis of anterograde and retrograde blood flow velocities were used to determine LBF during the movement phase of PLM, using the blood flow equation previously described. Peak LBF was calculated as the maximal value achieved during the first 30 sec of PLM. The change in LBF from baseline flow to peak flow (∆peakLBF) was calculated as peak LBF – baseline LBF. Cumulative area under the curve (AUC) for values of blood flow were determined and interpreted to indicate the overall increase in blood volume achieved during movement. AUC was calculated as the sum of LBF above baseline for each second during the 60‐sec movement phase of PLM, according to the trapezoidal rule and using the equation as follows: Σ(*y*_*i*_(*x*_(*i*+1)_ − *x*_*i*_) + (1/2)(*y*_(*i*+1)_  − *y*_*i*_)(*x*_(*i*+1)_ − *x*_*i*_)).

#### Flow‐mediated dilation analysis

End‐diastolic electrocardiogram (ECG) R‐wave gated images were collected from the video output of the Logiq e for offline analysis of brachial artery vasodilation using automated edge‐detection software (Medical Imaging Applications, Coralville, IA). %FMD was quantified as the maximal percentage in brachial artery diameter change following cuff release. Shear rate was calculated as: shear rate = 8*V*_mean_/arterial diameter. Blood flow was calculated as previously described.

### Assays

Blood samples were obtained through the antecubital vein at the time of the study visit. A portion of each sample was centrifuged at 1734 *g* for 10 min at 4°C. Serum from each sample was extracted and frozen at −80°C until analysis. Lipid oxidation, a marker of oxidative stress, was assessed by quantifying concentrations of plasma malondialdehyde (MDA) via an ELISA (LifeSpan BioSciences Inc., Seattle WA). Asymmetric dimethylarginine (ADMA), a metabolic byproduct known to inhibit NO synthesis and elevate oxidative stress, was also assessed by an ELISA (Eagle BioSciences Inc., Nashua, NH).

### Statistical analysis

Group differences in participant characteristics and vascular function measurements were analyzed using independent samples *t*‐tests. Additionally, relationships between participant characteristics and vascular function variables were analyzed using Pearson's *r* correlations. All analyses were performed using the Statistical Package for the Social Sciences (SPSS version 24, IBM, NY). Statistical significance was set at *α* ≤ 0.05. All data are presented as mean ± standard error of the mean (SEM).

## Results

### Participants

Participant characteristics and disease‐specific characteristics are displayed in Table 1. CKD patients and healthy controls were well matched for age and most of the characteristics assessed. CKD patients reported significantly higher BMI levels compared to healthy controls (30.7 ± 2 vs. 25.3 ± 1, respectively; *P* < 0.05), however, body fat percentage values were not different between the groups (33.0 ± 4 vs. 31.0 ± 2, respectively). Although within the normal range, CKD patients reported significantly lower high‐density lipoprotein levels compared to CON (*P* < 0.05). Additional hematology and biochemical parameters were within expected ranges for CKD patients and healthy controls (Inker et al. 2014). CKD status was confirmed in patients by a significantly lower eGFR (45.0 ± 6 vs. 88.9 ± 3 mL/kg/1.73 m^2^, *P* < 0.05) in the CKD patients when compared to controls. Demographics for both groups were representative of the state of Delaware population (U.S. Census Bureau, 2018).

**Table 1:** Participant characteristics

	CON	CKD
*n*	16	12
Demographics		
Age (years)	60 ± 2	66 ± 3
Sex (male/female)	6/10	7/5
Race (C/AA/A)	15/0/1	7/5/0
Body mass index (kg/m^2^)	25.3 ± 1	30.7 ± 2*
Body fat (%)	31.0 ± 2	33.0 ± 4
Resting hemodynamics		
Heart rate (beats per minute)	62 ± 2	68 ± 3
Systolic blood pressure (mmHg)	129 ± 3	145 ± 4*
Diastolic blood pressure (mmHg)	82 ± 1	86 ± 2
Mean arterial pressure (mmHg)	98 ± 2	106 ± 2*
Hematology and biochemistry		
eGFR (mL/kg/1.73 m^3^)	>60	45 ± 6*
Creatinine (mg/dL)	0.79 ± 0	1.75 ± 0*
Fasting glucose (mg/dL)	93 ± 2	100 ± 4
Total cholesterol (mg/dL)	197 ± 9	172 ± 14
HDL (mg/dL)	74 ± 5	53 ± 5*
LDL (mg/dL)	107 ± 7	102 ± 11
Hemoglobin (g/dL)	13.3 ± 0	12.4 ± 0*
Medications (*n*)		
Antihypertensive	1/16†	10/12
Antidiabetic	0/16	6/12
Statin	6/16	4/12

All values are mean ± SEM; CKD, chronic kidney disease; CON, healthy controls; C, Caucasian; AA, African American; A, Asian; eGFR, estimated glomerular filtration rate; HDL, high‐density lipoprotein; LDL, low‐density lipoprotein; *P ≤ 0.05, ^†^One subject was prescribed a medication classified as a diuretic, however, this medication was prescribed for dermatological purposes and this subject had no history of diagnosed hypertension.

### Peripheral responses to passive leg movement

**Figure 1:**
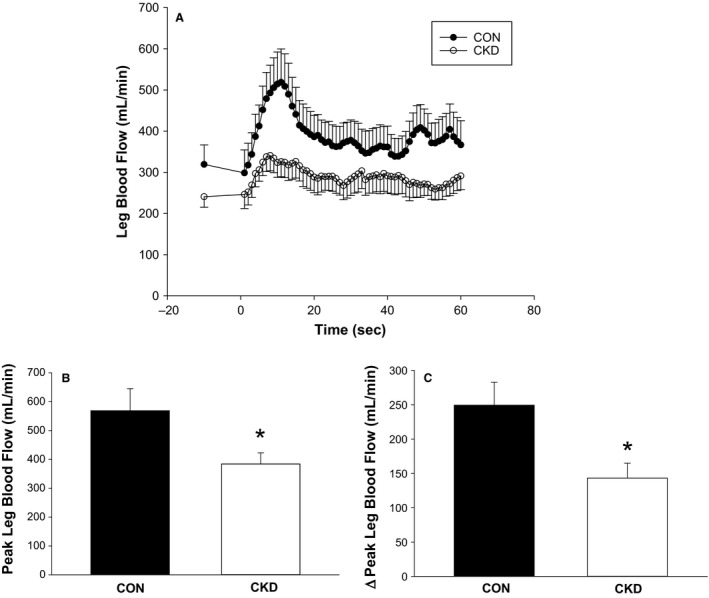
Hyperemia induced by passive leg movement (PLM). Second‐by‐second average LBF responses during PLM for CKD patients and healthy controls (CON) (A). Note: Figure A illustrates general blood flow trends that occurred during PLM. As the analyses were performed on data from individuals who exhibited varying response kinetics, second‐by‐second averaging removes some of the information obtained in individual recordings. Therefore, mean values for peak LBF (B) and ∆peakLBF (C) achieved within each group are also illustrated separately. LBF, leg blood flow; ∆peakLBF, change in leg blood flow from baseline to peak; CON, healthy controls; CKD, chronic kidney disease; *P < 0.05.

Second‐by‐second leg blood flow (LBF) responses to PLM for both CKD patients and healthy controls are illustrated in Figure 1A. There were no differences between the CKD patients and healthy controls baseline femoral artery diameters (0.86 ± 0.06 vs. 0.91 ± 0.05 mm, respectively) and baseline LBF (241 ± 25 mL/min vs. 319 ± 47 mL/min, respectively). PLM evoked a hyperemic response in both groups, however, peak LBF was significantly attenuated in CKD compared to healthy controls (384 ± 39 vs. 569 ± 77 mL/min, respectively; *P* < 0.05; Fig. 1B). In CKD patients, the change in LBF from baseline flow to peak flow (∆peakLBF) was also significantly blunted (143 ± 22 vs. 249 ± 34 mL/min, *P* < 0.05; Fig. 1C). Although AUC responses during PLM were reduced in those with CKD, these values were not significantly different between groups (49.7 ± 16 vs. 71.4 ± 19 mL, *P* = 0.40).

**Figure 2:**
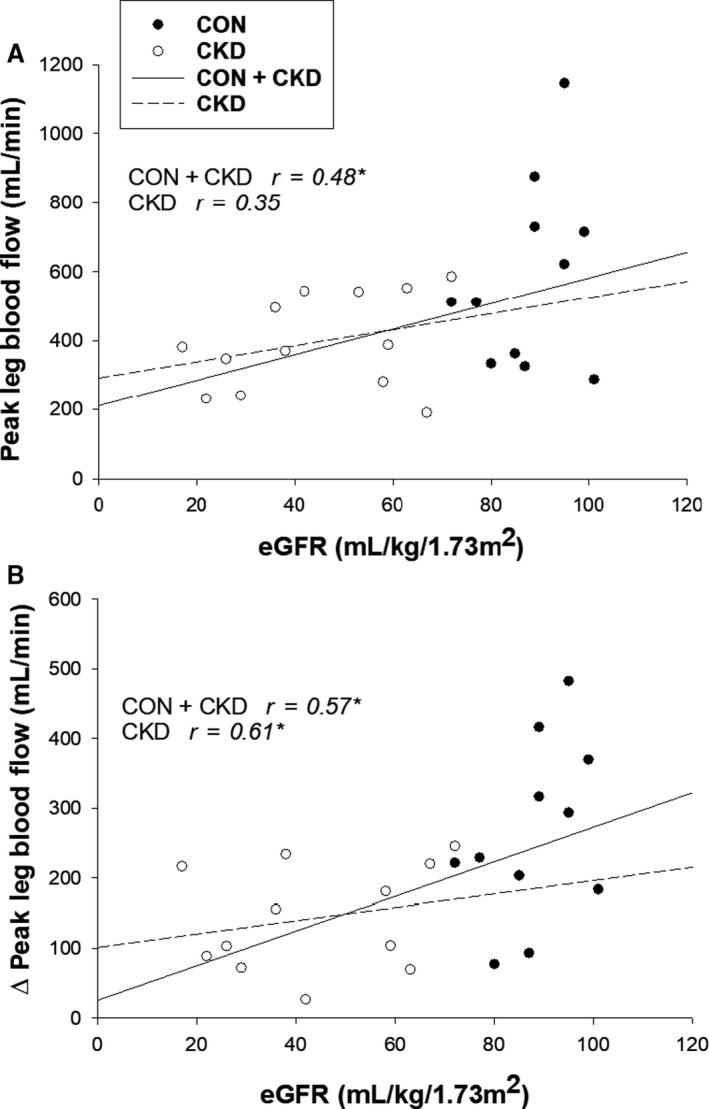
Associations between renal function and hyperemia evoked by passive leg movement (PLM). Renal function as indicated by eGFR was significantly associated with peak LBF (A) and ∆peakLBF (B) achieved during PLM in all subjects (solid line). Correlations between PLM‐induced hyperemia and eGFR for only patients with CKD are also presented (dashed line). eGFR, estimated glomerular filtration rate; LBF, leg blood flow; ∆peakLBF, change in leg blood flow from baseline to peak; *P < 0.05.

Associations between kidney function and PLM‐induced hyperemic responses were significant, such that eGFR was correlated with peak LBF (*r* = 0.41, *P* < 0.05) and ∆peakLBF (*r* = 0.50, *P* < 0.05) achieved during PLM. Results were similar when analyses were performed on only patients with CKD, where there was a trend between eGFR and peak LBF (*r* = 0.35, *P* = 0.27) and a significant association between eGFR and ∆peakLBF (*r* = 0.61, *P* < 0.05) (Fig. 2A and B).

### Brachial artery flow‐mediated dilation

**Figure 3:**
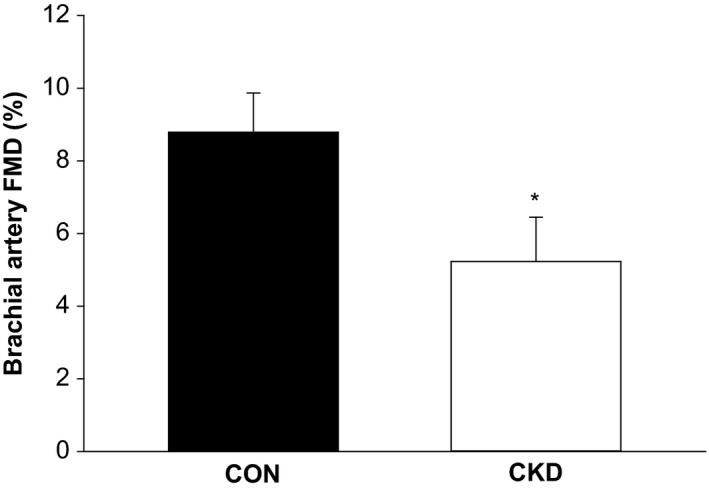
Brachial artery responsiveness to flow‐mediated dilation (FMD). Brachial artery responsiveness, as quantified by the maximal percent change in diameter from baseline (%FMD) during the 2 min immediately following the release of cuff occlusion, was significantly reduced in CKD patients when compared to controls. CON, healthy controls; CKD, chronic kidney disease; *P < 0.05.

Brachial artery diameters at baseline were not different in patients with CKD compared to controls (4.74 ± 0.3 vs. 4.11 ± 0.2 mm, respectively). Upper limb peripheral vascular function as measured by the percent change in brachial artery diameter from baseline to maximal dilation (%FMD) was significantly reduced in CKD patients (5.2 ± 1.1 vs. 8.8 ± 1.2%, *P* < 0.05; Fig. 3). There were no significant differences in the sum shear value up to the time of peak dilation between CKD patients and controls (39615 ± 4601 vs. 45210 ± 6039 sec^−1^, respectively; *P* = 0.49), therefore we did not normalize our FMD values for shear stress. Additionally, a significant relationship between kidney function as assessed by eGFR and FMD was observed for all participants for both %FMD (*r* = 0.48; *P* < 0.05) and ∆FMD (*r* = 0.46; *P* < 0.05).

### Physical activity assessment

**Figure 4:**
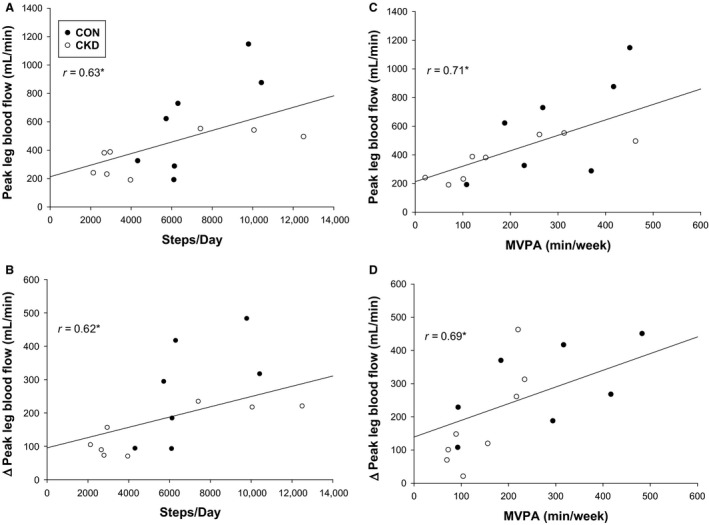
Associations between habitual physical activity and hyperemia evoked by passive leg movement (PLM). Habitual physical activity as expressed in average steps per day was significantly associated with both peak LBF (A) and ∆peakLBF (B) for all participants. Moderate‐to‐vigorous intensity physical activity (MVPA) as measured in minutes per week was also correlated with peak LBF (C) and ∆peakLBF (D). BF, leg blood flow; ∆peakLBF, change in leg blood flow from baseline to peak; CON, healthy controls; CKD, chronic kidney disease; *P < 0.05.

Habitual physical activity levels assessed were not different between CKD patients and healthy controls (Table 2). Physical activity as assessed by average steps per day was significantly associated with peak LBF (*r* = 0.63, *P* < 0.05) and ∆peakLBF (*r* = 0.62, *P* < 0.05) achieved during PLM for all participants (Fig. 4A and B). Additionally, minutes per week of moderate‐to‐vigorous intensity physical activity was also significantly associated with peak LBF (*r* = 0.71, *P* < 0.05) and ∆peakLBF (*r* = 0.69, *P* < 0.05) (Fig. 4C and D). No relationships between FMD responses and habitual physical activity measures were significant.

**Table 2:** Measures of habitual physical activity

	CON (*n* = 7)	CKD (*n* = 8)
Steps/day	6975 ± 851	5564 ± 1396
Physical activity kcal/day	397 ± 73	273 ± 45
Sedentary bouts/day	14 ± 1	11 ± 1
Hours sedentary/week	78 ± 4	69 ± 6
MVPA mins/week	290 ± 48	187 ± 52

All values are mean ± SEM; CKD, chronic kidney disease; CON, healthy controls; MVPA, moderate‐to‐vigorous intensity physical activity.

### Blood analyses

**Figure 5:**
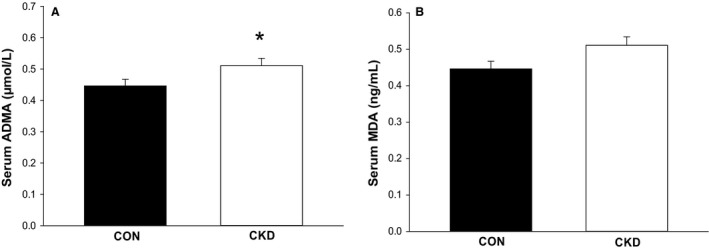
ADMA and MDA serum concentrations. ADMA concentrations were significantly higher in CKD patients when compared to controls (A). MDA concentrations were greater in CKD versus controls (B), however this difference was not statistically significant. ADMA, asymmetric dimethylarginine; MDA, malondialdehyde; CKD, chronic kidney disease, CON, healthy controls; *P < 0.05.

ADMA concentrations were significantly higher in patients with CKD when compared to controls (0.51 ± 0.02 vs. 0.45 ± 0.02 *μ*mol/L, respectively; *P* < 0.05) (Fig. 5A). ADMA concentrations were also significantly inversely associated with renal function of all subjects, as indicated by eGFR (*r* = −0.44, *P* < 0.05). MDA values also tended to be higher in patients with CKD when compared to controls (1732 ± 326 vs. 1493 ± 246 ng/mL, respectively; *P* = 0.56) (Fig. 5B), however, this difference was not close to significance.

## Discussion

This study sought to determine whether lower extremity vascular dysfunction, as assessed by PLM, is impaired in patients with CKD. As hypothesized, we observed a significantly diminished hyperemic response during PLM in CKD patients, suggesting impaired blood flow regulation and vascular dysfunction within the lower‐limb vasculature. We identified significant relationships between the hyperemic responses during PLM and kidney function, potentially providing insight on the dynamic relationship between kidney health, endothelial function, and risk of CVD development in older adults. Furthermore, we identified relationships between the magnitude of PLM hyperemia and habitual physical activity levels in all subjects. As anticipated, we found that brachial artery FMD was reduced in CKD, which is consistent with previous research. Taken together, these findings provide evidence of a widespread reduction in vascular responsiveness in patients with CKD which may be linked to the physical inactivity which is commonly reported in this population.

#### Passive leg movement and endothelial function

This study is the first of our knowledge to report a reduction in lower extremity vascular function as assessed by PLM in patients with CKD. We observed an attenuated hyperemic response during 1 minute of PLM in CKD patients compared to age‐matched healthy controls. Leg blood flow responses as assessed by peak leg blood flow were reduced by ~30% (Fig. 1B) and the increase in leg blood flow from resting values achieved during testing was ~40% lower in CKD patients compared to controls (Fig. 1C), indicating an inability to adequately perfuse the lower limb in response to the same stimulus applied in healthy older adults.

Endothelial dysfunction is an independent risk factor for future cardiovascular events (Widlansky et al. 2003) and the assessment of NO‐mediated vascular function is highly predictive of morbidity and mortality in various populations (Vallance and Chan 2001). PLM is a newer method to assessing vascular function in the lower extremity and evokes a hyperemic response that is mediated primarily by NO. PLM noninvasively assesses vascular health through quantification of blood flow at the femoral artery and indicates the function of downstream resistance vessels which are responsible for controlling perfusion of the locomotive muscles. The feasibility of PLM provides benefit beyond that of other noninvasive vascular assessments since it can detect easily‐identifiable changes in blood flow rather than small changes in vessel diameter, unlike that of FMD.

Mechanistically, at the onset of the passive movement of a limb, mechanical deformation of muscle fibers and connective tissues stimulates group III/IV mechanosensitive afferent nerve fibers and results in a hyperemic response to that region (McCord and Kaufman 2010). In its early application, Trinity et al. investigated the role of NO‐dependent vasodilation on reactive hyperemia during PLM in healthy adults and reported an 80% reduction in leg vascular conductance during L‐NMMA infusion (Trinity et al. 2012). Mortensen et al. also identified the contribution of NO during PLM hyperemia with reported associations between PLM responses and vasodilation via intra‐arterial infusions of acetylcholine (Mortensen et al. 2012). Previous investigations have utilized PLM to assess endothelial dysfunction in various populations and disease states which are characterized by reduced NO bioavailability, such as older adults (McDaniel et al. 2010; Groot et al. 2015), heart failure (Hayman et al. 2010; Witman et al. 2015), and sepsis patients (Nelson et al. 2016). These studies report responses similar to those in our current investigation and conclude that the reduced PLM responsiveness may be, in part, due to individual pathophysiological NO deficiencies.

We identified several associations between PLM responsiveness and kidney function in all subjects. We observed that those with a lower eGFR had lower peak leg blood flow values (Fig. 2A) and an overall reduced ability to increase their leg blood flow compared to those with a higher eGFR (Fig. 2B). The physiological relationship between vascular function and kidney function is likely both dynamic and cyclical such that poor kidney function negatively influences vascular structure and function, meanwhile attenuations in vascular function may also negatively impact the health of the kidney due to inadequate perfusion. The relationships identified between kidney and vascular function in this study suggest novel implications for PLM use, such as assessment of the responsiveness of the vasculature which is primarily responsible for perfusing the locomotive skeletal muscles needed for adequate physical activity levels. This is especially important in populations at risk for CVD development, such as patients diagnosed with CKD.

#### Endothelial function and physical activity

Another major finding of our study was the relationships identified between lower extremity vascular health and habitual physical activity levels in all subjects. Patients with CKD are known to be physically inactive and often have reduced cardiorespiratory fitness levels. It was recently reported that a standard group of stage 3–5 nondialysis CKD patients acquired only 6 ± 9 min of moderate‐to‐vigorous intensity physical activity per day, suggesting that these patients achieve far less than the national recommendations of 150 min per week (West et al. 2017). Similarly, significantly reduced VO_2_peak measurements have been reported in patients with CKD when compared to sedentary age‐predicted norms (Padilla et al. 2008). Alternatively, higher cardiorespiratory fitness levels and greater endothelial function are both indicative of lower CVD risk (Hadi et al. 2005; Myers et al. 2015), suggesting these factors as potential therapeutic targets for reducing CVD risk in those with CKD. We documented here that higher levels of habitual physical activity were associated with greater lower extremity vascular function as indicated by a greater magnitude of reactive hyperemia during PLM (Fig. 4A–D). This highlights the impaired regulation of blood flow to the primary skeletal muscles responsible for locomotion in those who are less physically active and it is likely that this may be contributing to the elevated risk of CVD in patients with CKD.

#### Flow‐mediated dilation in CKD

Brachial artery FMD is a traditional measurement of vascular function that is partially NO‐mediated and has been identified as an independent predictor of long‐term adverse cardiovascular events (Shechter et al. 2014). Previous investigations have identified endothelial dysfunction via attenuated FMD responses in varying stages of CKD, including those with mild‐to‐moderate and end‐stage kidney disease (Cross et al. 2003; Kuczmarski et al. 2011), similar to the blunted FMD responses indicated in the CKD patients from our study (Fig. 3A–B). Results from both FMD and PLM assessments can be interpreted in combination, indicating the likelihood of widespread NO‐deficiency and subsequent endothelial dysfunction within the conduit arteries and downstream vasculature in CKD which can be identified prior to any clinical CVD manifestation.

#### Possible mechanisms of endothelial dysfunction in CKD

Several potential mechanisms are hypothesized to be responsible for reduced NO bioavailability in patients with CKD and potentially responsible for the reduced PLM and FMD responses reported here. Oxidative stress, defined as an imbalance between free radical production and removal by endogenous antioxidants (Guzik and Harrison 2006), is a known contributor to endothelial dysfunction through its ability to impair NO signaling in endothelial cells. Oxidative stress has been documented in patients with varying stages of moderate‐to‐severe CKD including those without diagnosed CVD (Kirkman et al. 2018). Although our values did not reach significance, patients with CKD displayed higher values of circulating malondialdehyde (MDA) than controls (Fig. 5), consistent with previous literature (Agarwal 2004; Xu et al. 2015). It is possible that elevated oxidative stress levels in our CKD patients may have contributed to the reduced endothelial function identified in this study.

NO production within endothelial cells relies on synthesis of L‐arginine, however, production of L‐arginine is reduced in CKD due to a loss of functional renal tissue (Baylis 2008). In addition, accumulation of uremic toxins seen in CKD potentially impairs L‐arginine transport and subsequently reduces its availability for NO synthesis (Martens and Edwards 2011). Asymmetric dimethylarginine (ADMA) is a uremic toxin which is known to circulate in higher amounts in CKD (Vallance et al. 1992; DuPont et al. 2014). Elevated ADMA levels result in reduced NO production through inhibition of nitric oxide synthase (NOS) and possibly contributes to the impaired vasorelaxation, elevated inflammation, and reduced angiogenesis identified in CKD (Sibal et al. 2010). As anticipated, we identified significantly greater circulating concentrations of ADMA in our patients with CKD when compared to controls (Fig. 5). Although these values were not significantly associated with PLM‐induced hyperemia, we did identify an inverse trend between ADMA and PLM hyperemia when represented as area under the curve (*r* = −0.35, *P* = 0.08, data not shown). Further investigation is necessary to identify the role that ADMA might play in the blunted PLM responses identified in patients with CKD.

#### Study limitations

We acknowledge several limitations which should be considered when interpreting the results of this study. First, while brachial artery FMD was utilized to help validate our PLM findings, we recognize that a lower extremity FMD would be more appropriate for validation of localized lower‐limb vascular dysfunction. Nonetheless, brachial artery FMD still provides important information which can be interpreted in combination with the results of PLM. From a mechanistic perspective, FMD provides information about localized, large conduit artery function. Alternatively, the hyperemic response during PLM is likely influenced substantially by the responsiveness of the microvasculature, providing us with additional information on downstream function. In addition, our goal was to characterize potential limb‐specific impairments in vascular function and using measures of both upper extremity and lower extremity vascular measures allowed us to highlight this. Second, there are known limitations to using bioelectrical impedance analysis (BIA) in renal populations due to potential fluid shifts and elevated extracellular volume. These fluid disturbances that may be associated with inaccurate BIA results are often reported in dialysis patients (Kushner et al. 1996; Arkouche et al. 1997; Kamimura et al. 2003), however, the current study excluded CKD patients on dialysis. Moreover, BIA was recently shown to accurately estimate body composition against dual x‐ray absorptiometry, the method recommended by clinical practice guidelines for nutrition in chronic renal failure (Kopple 2001), in a group of nondialysis CKD patients similar to those recruited for our study (Wilkinson et al. 2019). Finally, we recognize that our participants with CKD also had a high incidence of comorbidities, which may be contributing to their vascular dysfunction, and thus, the independent effect of reduced renal function on our vascular measurements cannot be concluded. Nonetheless, we believe there is substantial scientific and clinical value in assessing vascular function in patients of a realistic disease status, as it still highlights important observations that are representative of the larger community of patients with CKD.

## Conclusion

Through our use of peripheral vascular assessments in both the upper and lower extremities, we provide evidence of widespread vascular dysfunction in patients with CKD. This is likely mediated, at least in part, by a reduction in NO bioavailability which has been previously documented in this population. This study uses PLM as a novel method in patients with CKD and provides evidence of dysfunction of the vasculature which perfuses the locomotive muscles. It is possible that the vascular dysfunction is contributing to exercise intolerance and reduced cardiorespiratory fitness levels characteristic of patients with CKD, which is associated with a higher CVD burden in this population (Howden et al. 2015). Results from this study indicate the need for future investigations examining the mechanisms linking lower extremity vascular function with physical activity levels, which could be mediating some of the risk associating CVD with CKD.

## Conflict of Interest

None declared.
